# Efficacy of toothpaste containing OPTIMEALTH® OR in inhibiting dental plaque and gingivitis: A randomized controlled trial

**DOI:** 10.1097/MD.0000000000041225

**Published:** 2025-01-31

**Authors:** Yimin Fu, Yong Yang, Keyun Mu, Yuye Zhou, Hui Chai

**Affiliations:** a Zhejiang Chinese Medical University, Hangzhou, People’s Republic of China; b Sethic (Guangzhou) Research & Development Center Co., Ltd, Guangzhou, People’s Republic of China.

**Keywords:** bleeding on probe, gingival index, gingivitis, microbiota, OPTIMEALTH® OR, plaque index

## Abstract

**Background:**

This randomized double-blind, placebo-controlled clinical trial evaluated the effects of 2% OPTIMEALTH® OR toothpaste in regulating dental plaque microbiota and alleviating gingivitis.

**Methods:**

Subjects were randomly assigned to the placebo group and test group. They were instructed to brush their teeth with placebo toothpaste (placebo group) or OPTIMEALTH® OR toothpaste (test group) for a continuous 4 weeks. Clinical indices of plaque index, gingival index, and bleeding on probe (%) were examined, and images of dental plaque staining were captured at baseline and after 2 and 4 weeks. The plaque microbiome was analyzed by 16s rDNA amplicon sequencing at baseline and after 4 weeks.

**Results:**

Thirty-two participants with similar characteristics were recruited. After using OPTIMEALTH® OR toothpaste for 4 weeks, a decrease of 27.05% (*P* < .01), 8.29% (*P* > .05), and 47.44% (*P* < .05) in plaque index, gingival index, and bleeding on probe (%) scores was observed compared to the baseline, respectively. The extent of decline in these indices is greater than that in the placebo group. A decrease in dental plaque could be observed after 2 and 4 weeks in the test group. The 16s rDNA sequencing results showed that the observed species index and Chao index, but not the Shannon index and beta diversity, were reduced significantly after using OPTIMEALTH® OR toothpaste for 4 weeks. In addition, compared with the placebo group, using OPTIMEALTH® OR toothpaste reduced the abundance of bacterial species such as *Veillonella parvula* and *Prevotella denticola*.

**Conclusion:**

Brushing teeth with 2% OPTIMEALTH® OR-fortified toothpaste could effectively reduce dental plaque and regulate plaque microbiota.

## 1. Introduction

Gingivitis, commonly known as dental plaque-induced gingivitis, is defined as a localized inflammatory condition initiated by the accumulation of dental biofilm.^[1,2]^ It is responsible for the majority of cases of gingival inflammation.^[3]^ As one of the most common human inflammatory diseases, dental plaque-induced gingivitis is ubiquitous throughout the globe, irrespective of age, gender, or race.^[4]^ Untreated gingivitis is a risk factor for periodontitis, a chronic oral inflammatory disease.^[5]^ Nonetheless, in contrast to periodontitis, one distinctive feature of plaque-induced gingivitis is the complete reversibility of tissue changes once the dental biofilm is eliminated.^[6]^ Therefore, the management of gingivitis is a crucial primary prevention strategy for periodontitis and a secondary prevention strategy for its recurrence.^[7]^ Today, brushing teeth remains the primary prevention and management strategy for gingivitis. To enhance the efficacy of removing dental plaque, some studies have attempted to incorporate antibacterial ingredients or herbal extracts into toothpaste.^[8–10]^

Recent studies are showing a growing recognition of the significant influence of the oral microbiome on gingivitis.^[11]^ The oral microbiome commonly exists in the form of biofilms.^[12]^ The oral bacteriota, along with commensal fungi, form functionally organized, multi-species biofilms and establish a network of intricate mutual interactions between different species, fostering a symbiotic relationship between microbes and the host to maintain oral and systemic health in humans.^[12]^ It has been proved that probiotics, prebiotics, and postbiotics modulate the oral biofilm composition or control plaque.^[12–14]^ Many clinical trials have focused on the beneficial effects of probiotics on gingival health. The consumption of a probiotic combination of *Lactobacillus rhamnosus* and *Bifidobacterium lactis* improved the gingival health in adolescents.^[15]^ Supplementing with probiotic yogurt containing *Bifidobacterium animalis* can positively impact both plaque accumulation and gingival inflammatory parameters.^[16]^ However, few clinical trials mentioned the postbiotic in improving gingival health.

OPTIMEALTH® OR (INCI: Water, Lactobacillus/Soymilk Ferment Filtrate, Butylene Glycol, Pentylene Glycol) is a postbiotic, produced by co-fermentation of 16 types of lactic acid bacteria (*Bifidobacterium longum*, *Bifidobacterium adolescentis*, *Lactobacillus acidophilus*, *Levilactobacillus brevis*, *Lactobacillus jensenii*, *Lactobacillus gasseri*, *Lactococcus lactis*, and so on), with black beans as substrate. This fermented product does not contain the living bacteria but retains bacterial lysates, and more than 300 active molecules have been identified and quantified, including organic acids, bacteriocins, peptides, short-chain fatty acid, neurotransmitters, phospholipids, vitamins, flavonoids, isoflavones, lipoteichoic acid, exopolysaccharides, and so on. Many of these bacterial metabolites and bacterial lysates have shown beneficial effects on human health. For example, bacteriocins regulate competitive interactions within the microbial community, enhancing gut barrier function and host immune response, offering an alternative or supplement to antibiotics^[17]^; short-chain fatty acid, derived from the microbial fermentation of indigestible carbohydrates in the gut, are crucial for maintaining gastrointestinal and metabolic health^[18]^; flavonoids, abundantly found in vegetables, fruits, cereals, nuts, and herbs, are well-known for their anti-inflammatory effects in human bodies^[19]^; exopolysaccharides from lactic acid bacteria are recognized for their antioxidant, antiviral, antitumor, and immunomodulatory properties.^[20]^ Nevertheless, clinical trials are still needed to validate the efficacy of OPTIMEALTH® OR on oral health.

It can be assumed that brushing teeth with the toothpaste containing OPTIMEALTH® OR could regulate the plaque microbiome and alleviate gingivitis. This study aimed to evaluate the effect of OPTIMEALTH® OR toothpaste on the clinical oral inflammatory status and dental plaque microbiome regulation.

## 2. Materials and methods

### 2.1. Study design

This is a prospective, double-blind, randomized, 2-arm, parallel-group, placebo-controlled study with an intervention period of 4 weeks. Data were collected between December 2022 and January 2023 at Hospital of the Stomatology Xi’an Jiaotong University. All the subjects were subjected to a 1-week washout period, and the assigned toothpastes were continuously used for 4 weeks. An oral examination and sample collection were performed at baseline and then after 2 and 4 weeks. Subjects and examiners were blinded to treatment assignment for the duration of the study. The study was approved by the Ethics Committee of Shaanxi Biocell General Testing Co, Ltd (BXLL2022033).

### 2.2. Sample size

Based on the differences observed in the primary outcome from a preliminary study involving 46 diagnosed periodontitis patients, the required sample size was determined.^[21]^ The calculation was performed using G*Power 3.1.9.6 software (HHU, Düsseldorf, Germany). The mean gingival index (GI) score for the placebo control group was 57.2 ± 37.0, and the GI score for the proanthocyanidins treatment group was expected to decrease by 46.6 points. With a type I error (α) set at 0.05 and power set at 0.90, the sample size was calculated to be 28 patients according to the sample size calculation formula: $n=\frac{2{\left(Z\alpha +Z\beta \right)}^{2}*\sigma^{2}}{\delta^{2}}$. Accounting for a 10% data loss, the total required sample size was determined to be 32 participants.

### 2.3. Subject

**Figure 1.**
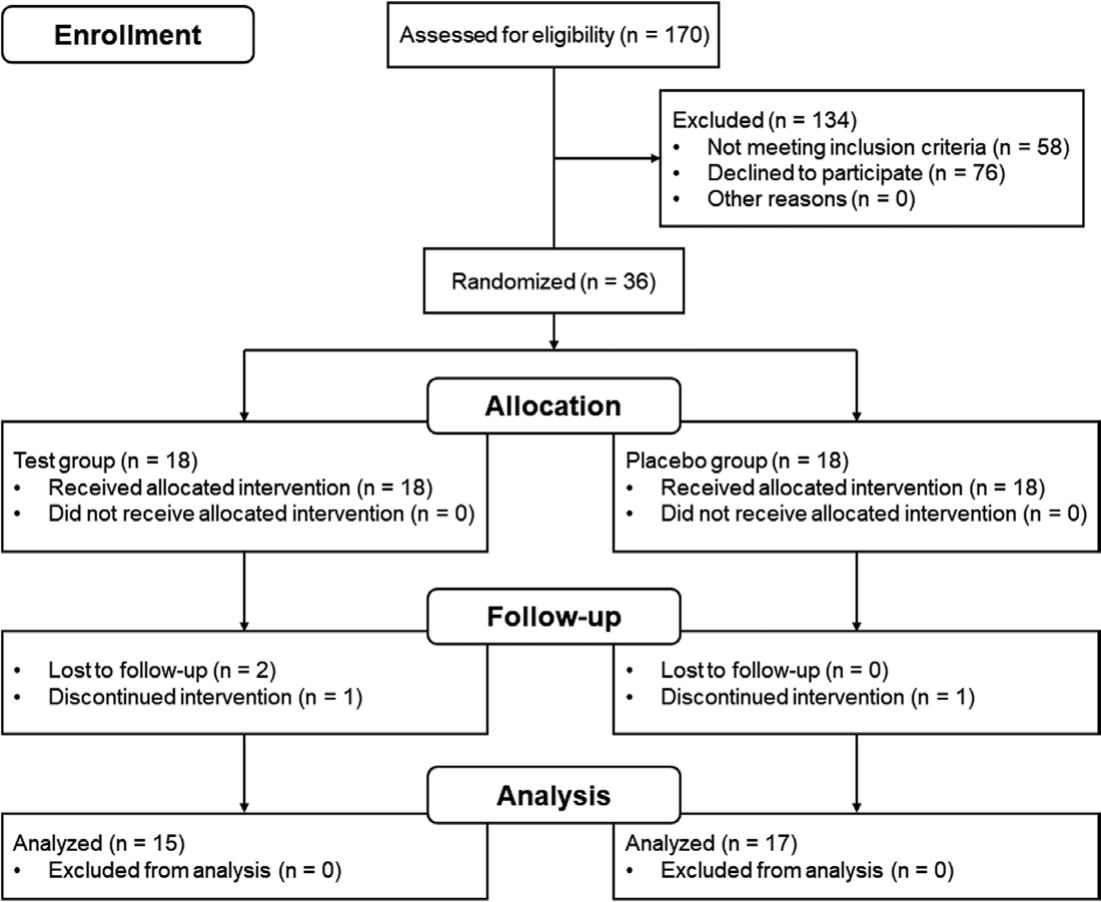
Subject flow during the trial.

Recruitment was conducted by social media and flyers. A total of 170 Asian participants were assessed for eligibility to be included in the study, and 32 overall healthy subjects (aged between 23 and 60 years, with an average age of 40.16 ± 10.08 years, 24 females) participated in and completed this study (Fig. 1). Informed consents were obtained from all participants.

Inclusion criteria were as follows: the age range is 18 to -60 years old, and the age range is evenly distributed, regardless of gender; good overall health status and no significant systemic diseases; having more than 20 natural teeth; the average plaque index (PI) score of not <10 teeth is ≥1.5; and not participating in other similar experimental studies at the same time.

Exclusion criteria were as follows: wearing orthodontic braces; wearing orthodontic appliances or removable partial dentures; pregnant or lactating women; open caries or mucosal lesions in the oral cavity; untreated caries lesions (excluding mild caries), mucosal diseases, and pericoronitis; have a history of using antibiotics 1 month before entering the trial; patients with diabetes, heart disease, and Alzheimer disease; individuals with a history or sensitivity to toothpaste; subjects have received oral cosmetic care within 1 week; subjects undergoing clinical trials or medical care of other oral samples within 1 month; has undergone oral treatment in the past month or is currently in the recovery phase (such as root canal therapy and porcelain veneers); and other situations that dentists consider unsuitable for trial use.

### 2.4. Intervention and placebo control

Subjects who met the inclusion criteria were randomized 1:1 to receive either placebo toothpaste (blank matrix toothpaste, a kind of experimental non-sodium bicarbonate, silica sodium fluoride toothpaste; placebo group; n = 15) or the same toothpaste but containing 2% (w/w) OPTIMEALTH® OR (test group; n = 17). The toothpastes were supplied by Sethic (Guangzhou) Research & Development Center Co, Ltd (Guangzhou, China), and the preparation of toothpaste had been done by the toothpaste producer in the preliminary stage, which is strictly in accordance with the Chinese Pharmacopoeia and the industry standards.

### 2.5. Study protocol and restrictions

At the washout period of 7 days, subjects were instructed to use blank matrix toothpaste. After that, subjects were instructed to brush their teeth using assigned toothpaste twice a day, with each brushing session lasting at least 2 minutes. The amount of toothpaste used per brushing was equivalent to the size of 1 pea. The Bass Method was employed for tooth brushing. The trial lasted for 4 consecutive weeks. The subjects were instructed to undergo oral examination and/or sample collection between 9 am and 12 am on the 1st (week 0), 15th (week 2), and 29th (week 4) day. At week 0 (baseline) before the assigned toothpaste was used, as well as at week 2 and week 4 after the assigned toothpaste was used, the PI, the GI, and the bleeding on probe (BOP) score index (%) of the subjects’ teeth (lip side) were examined by a dental practitioner. In addition, samples were collected and identified for 16s rDNA in both supragingival and subgingival plaques (mixed into 1 tube) at week 0 and week 4.

Restrictions on subject were as follows: during the trial period, the subjects did not use oral care samples and drugs such as mouthwash, dental powder, dental patches, and teeth washing, nor did they receive any oral cosmetic medical care; do not drink water within 1 hour before sampling; do not take or apply antibiotics or other drugs that affect the microbiota during the testing period; the water temperature of the mouthwash used by volunteers during the use of the sample should be consistent when brushing their teeth; the subjects did not eat after brushing their teeth at 8 pm the day before the test, and the follow-up test was scheduled from 9 am to 12 am the next day. They were also not allowed to eat or brush their teeth before the follow-up test; subjects should avoid drinking alcohol 24 hours before testing; and subjects should avoid all oral health measures and chewing gingival consumption 12 hours before testing.

### 2.6. Randomization and allocation concealment

Each subject was given a code number at the enrollment visit, and they were assigned randomly to 1 of the 2 groups based on a computer-generated table (conducted by investigator Fu Yimin). According to the generated sequence, the tubes of the 2 types of toothpaste were printed with code number of each subject. Investigator Yang Yong was responsible for recruiting participants and distributing toothpaste according to their code numbers. The random allocation sequence was only revealed after the end of the trial, and prior to this, any investigator and examiner other than Fu Yimin were unaware of the allocation.

### 2.7. Examination parameters

Using the PI (Turesky et al modified Quigley-Hein PI)^[22,23]^ to check and record the plaque coverage area on the cheek and tongue surfaces of the index teeth. Using the GI^[24]^ to record gingival inflammation. Using the percentage of bleeding sites in the total site of the mouth (BOP%) to record and divide the degree of gingival health. For dental plaque imaging and analysis, subjects wore a dental mouth opener, and VISIA7 (Canfield, USA) was utilized to capture images of their lips and teeth with plaque staining.

### 2.8. DNA isolation and sequencing

Dental plaque microbiome DNA was isolated with the TIANamp Swab DNA Kit (DP322-03, Chengdu Feike Biotech, China), then amplified via PCR with primers 338F (5′-CCTACGGGAGGCAGCAG-3′)/806R (5′-GGACTACNNGGTATCTAAT-3′) to obtain partial bacterial 16S rDNA. The amplifying condition was activating at 95°C for 5 minutes, followed by 28 cycles of 45 seconds at 95°C, 50 seconds at 55°C, and 45 seconds at 72°C, then followed by 10 minutes at 72°C, and finalized by a 10-minute extension at 72°C. The amplified products were used to construct a microbial diversity sequencing library with the NEB Next Ultra II DNA Library Prep Kit (New England Biolabs, Inc, USA) and then sequenced in Beijing Ovison Gene Technology Co, Ltd (Beijing, China) using the Illumina Miseq PE300 high-throughput sequencing platform. Metagenomic analysis was carried out on the online platform OmicStudio tools (https://www.omicstudio.cn/tool).

### 2.9. Statistical analysis

The data were expressed as means ± standard deviation or means ± standard error of the mean. A 2-tailed unpaired *t* test was used for statistical significance testing of clinical indicators and relative abundance. The Kruskal-Wallis statistical test was used for statistical significance testing of alpha diversities. The data analysis was conducted using the SPSS statistical software, version 22.0. The statistical significance is set at *P* value < .05.

## 3. Results

### 3.1. Baseline characteristics

The subject baseline values, including age, sex, PI, GI, and BOP%, were similar to those of the placebos (*P* > .05; Table 1). The compliance of the subjects was generally good; however, 2 of them did not meet the study criteria during the follow-up period, and 2 were lost to follow-up (Fig. 1). Each volunteer used the test sample as required, and no obvious adverse events were recorded during the trial.

**Table 1:** Baseline demographic and clinical characteristics.

	Placebo group (n = 15)	Test group (n = 17)
Male gender, n (%)	3 (20)	5 (29.41)
Race, n (%)		
Asian	15 (100)	17 (100)
Age, yr, mean (SD)	39.93 (10.87)	40.35 (9.67)
Smoking, n (%)	0 (0)	0 (0)
PI, mean (SD)	2.58 (0.28)	2.59 (0.29)
GI, mean (SD)	1.25 (0.37)	1.28 (0.27)
BOP%, mean (SD)	14.52 (14.94)	15.38 (11.83)

BOP = bleeding on probe, GI = gingival index, PI = plaque index, SD = standard deviation.

### 3.2. PI, GI, and BOP%

**Figure 2.**
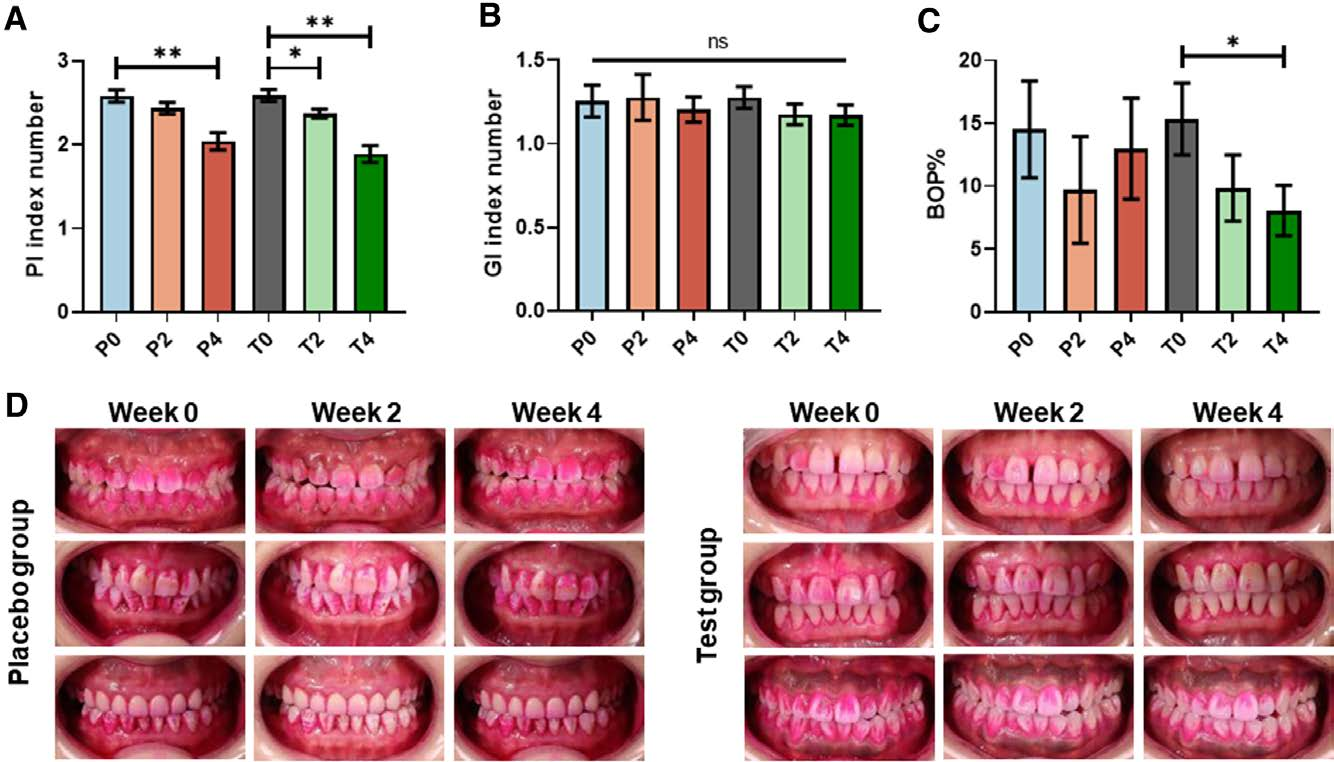
Clinical indices and plaque staining. PI (A), GI (B), and BOP% (C) during the trial (means ± SEM). Representative dental plaque staining photography (D). P0/2/4, placebo group at baseline/week 2/week 4; T0/2/4, test group at baseline/week 2/week 4. The *P* values were calculated using 2-tailed unpaired *t* test. *P < .05, **P < .01. BOP = bleeding on probe, GI = gingival index, PI = plaque index, SEM = standard error of the mean.

Both the placebo toothpaste and the OPTIMEALTH® OR toothpaste showed statistically significant reductions in PI score compared to baseline values after 4 weeks (Fig. 2A). The placebo toothpaste reduced 5.68% and 20.97% of PI at week 2 and week 4, respectively, compared to baseline; simultaneously, the OPTIMEALTH® OR toothpaste achieved reductions of 8.41% and 27.05% (Fig. 2A). The images of plaque captured from subjects exhibited a consistent result (Fig. 2D), suggesting the positive effect of OPTIMEALTH® OR toothpaste on dental plaque removal.

The placebo toothpaste reduced −1.81% and 3.99% of GI at week 2 and week 4, respectively, compared to baseline (Fig. 2B). In comparison, the OPTIMEALTH® OR toothpaste achieved reductions of 7.83% and 8.29% (Fig. 2B). However, these observed changes did not reach statistical significance (*P* > .05). Besides, no statistically significant differences were observed in the GI score between the test group and the placebo group (*P *> .05; Fig. 2B).

We also evaluated the impact of OPTIMEALTH® OR toothpaste on BOP% in participants. However, the placebo group exhibited BOP% reductions of 33.07% and 10.58% after 2 and 4 weeks, respectively. But, compared to the baseline, no statistical significances were obtained (*P* > .05; Fig. 2C). While the test group resulted in reductions of 35.77% and 47.44% at the same time points, the last 1 exhibited a statistically significant change (Fig. 2C).

Collectively, we observed a notable decrease in the clinical indices (PI and BOP%) among the subjects who were using the OPTIMEALTH® OR toothpaste.

### 3.3. 16s sDNA sequencing

**Figure 3.**
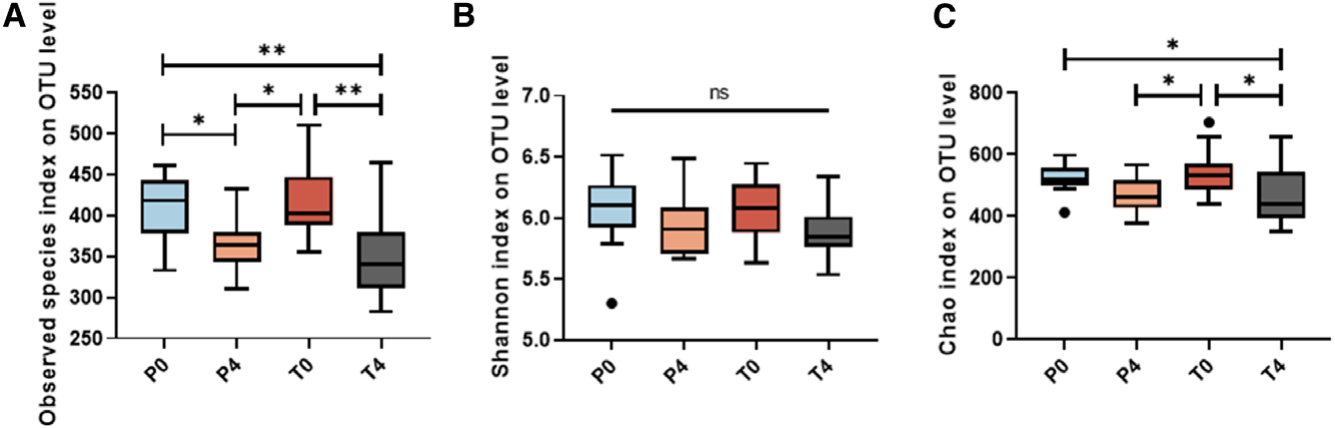
Differences in microbial diversity according to the observed species index (A), Shannon index (B), and Chao index (C). The *P* values were calculated using Kruskal-Wallis statistical test. *P < .05, **P < .01. OTU = operational taxonomic units.

Three alpha diversity indices were employed to assess the microbial diversity within samples: observed species index (Fig. 3A), Shannon index (Fig. 3B), and Chao index (Fig. 3C). The placebo group revealed a reduction of observed species index (*P *< .05), while the other indices were not changed obviously (*P *> .05). Using OPTIMEALTH® OR toothpaste significantly reduced observed species index (*P *< .01) and Chao index (*P* < .05), but the Shannon index showed no statistical difference (*P* > .05). The results indicated that the use of OPTIMEALTH® toothpaste primarily reduced the abundance of bacteria.

**Figure 4.**
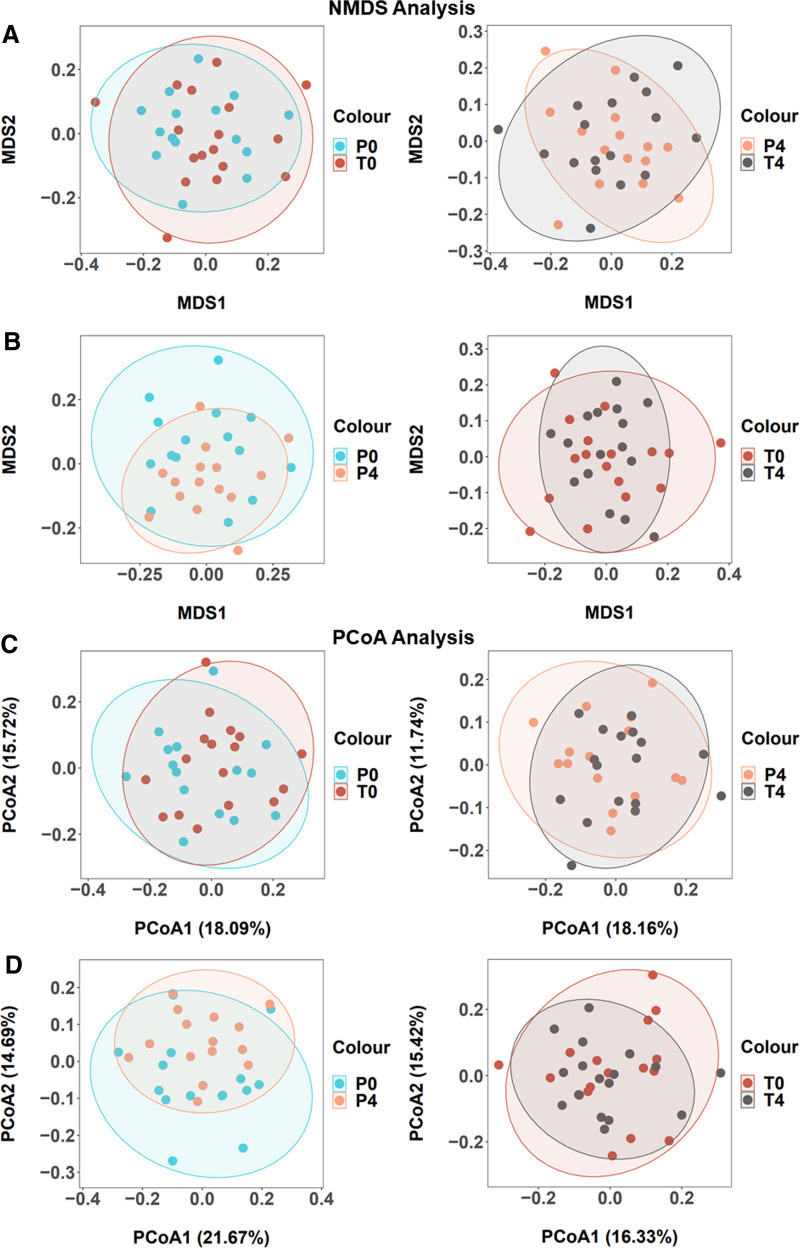
Non-metric multidimensional scaling (NMDS) ordinations and principal coordinates analysis (PCoA) ordinations by Bray-Curtis distances. Samples are colored according to their treatment (A, C) or respective time point (C, D). Ellipses indicate 95% confidence interval for the standard deviations of points in each group.

As visualized through non-metric multidimensional scaling using Bray-Curtis distances, no noticeable differences in the microbiome composition of plaque samples were observed between the placebo group and test group before and after follow-up (Fig. 4A). Compared to the baseline, both the placebo group and the test group samples did not exhibit separation but revealed a contraction in dispersion distance at week 4 (Fig. 4B). The samples were further analyzed using principal coordinates analysis for visualization (Fig. 4C, D). Consistent with the non-metric multidimensional scaling analysis mentioned earlier, similar patterns and trends were observed.

**Figure 5.**
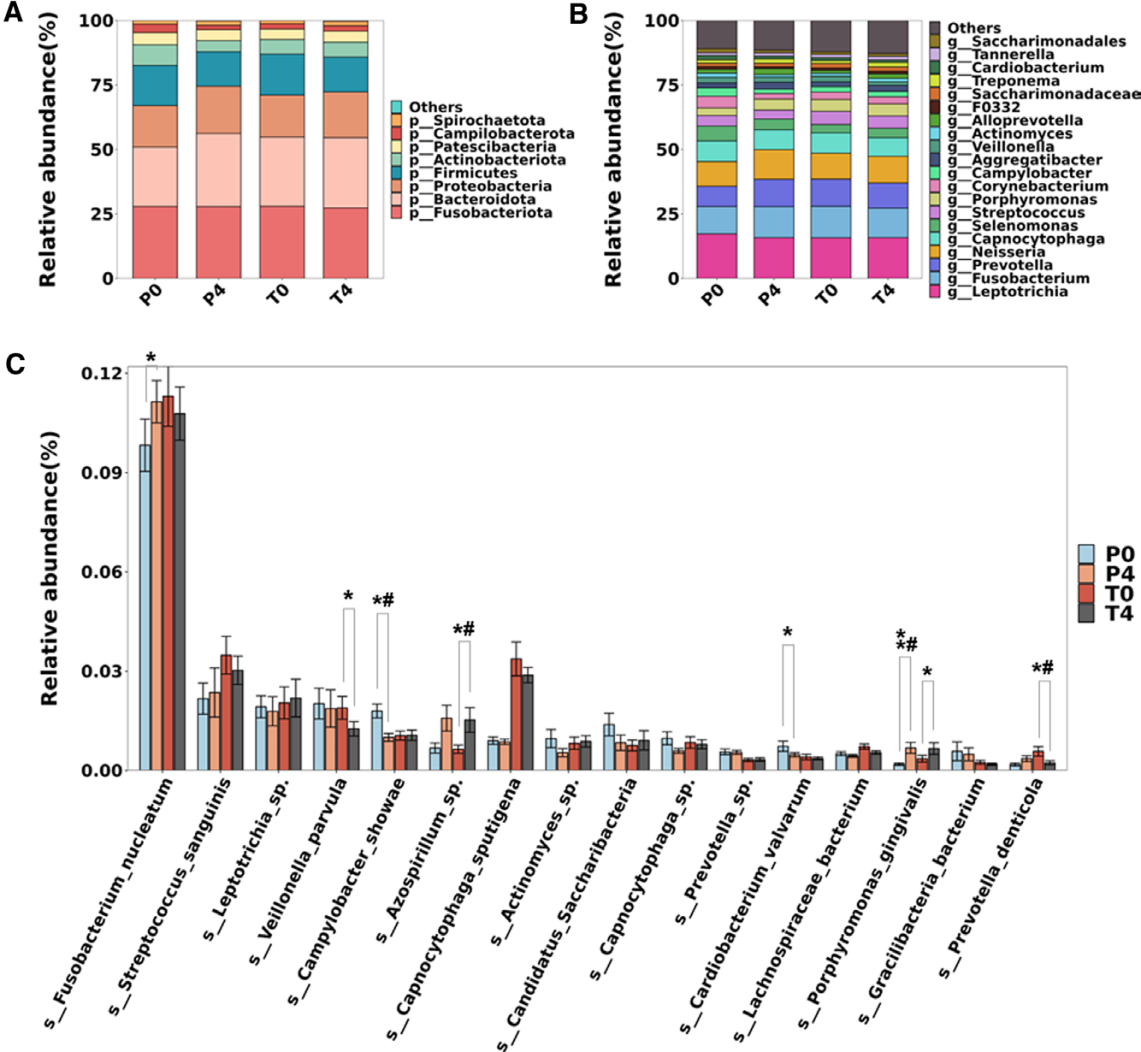
Composition of microbial communities across samples at the phylum, genus, and species levels. Relative abundance of the top 8 phyla (A). Relative abundance of the top 20 genera (B). Relative abundance of the top 16 species (C). P0/4, placebo group samples at baseline/week 4; P0/4, placebo group samples at baseline/week 4. The *P* values were calculated using 2-tailed t test. *P < .05, **P < .01 with paired t test. #*P* < .05 with unpaired *t* test.

The bacterial communities in the samples at baseline and week 4 were analyzed at the phylum, genus, and species levels (Fig. 5). *Fusobacteriota*, *Bacteroidota*, *Proteobacteria*, *Firmicutes*, *Actinobacteria*, and *Patescibacteria* dominated in all dental plaque samples. Their relative abundances show minimal differences among samples (Fig. 5A). At the genus level, *Leptotrichia*, *Fusobacterium*, *Prevotella*, and *Neisseria* were the 4 most abundant genera. However, the changes in the bacterial composition at the genus level were not obvious between the 2 groups after using their respective toothpastes (Fig. 5B). Among the high-abundance bacterial species, there was a significant reduction in the abundance of *Veillonella parvula* and *Prevotella denticola* after using the OPTIMEALTH® OR toothpaste compared to baseline. *Campylobacter showae* and *Cardiobacterium valvarum* showed a significant decrease in the placebo group, whereas *Fusobacterium nucleatum* increased compared to baseline. *Porphyromonas gingivalis* increased in both groups, but the increase was more pronounced in the placebo group (Fig. 5C).

## 4. Discussion

This 4-week double-blind randomized controlled trial was conducted to evaluate the clinical and oral microbial effects of brushing with a toothpaste containing 2% OPTIMEALTH® OR. Various indices, including PI, GI, and BOP, plaque staining, and the bacteriota of dental plaque, were examined. The study found that compared to the placebo group, the test group showed more improvements in PI, BOP, and plaque staining. Furthermore, alterations in the bacteriota composition of dental plaque were observed.

The prevalence of periodontal disease, which includes gingivitis and periodontitis, has experienced a substantial increase of 57.3% from 1990 to 2010.^[25]^ Notably, periodontitis affects more than half of the adult population worldwide.^[26]^ The primary risk factor for periodontitis is the accumulation of a plaque biofilm at and below the gingival margin, where dysbiosis emerges, triggering an inappropriate inflammatory immune response from the host.^[7]^ It should be noted that gingivitis acts as both a precursor to periodontitis and a reversible form of the disease, provided that a healthy oral hygiene status is reinstated.^[6,27]^ Therefore, actively controlling dental plaque is an effective measure for preventing periodontal disease. Previous studies have attempted to enhance the efficacy of toothpaste in alleviating gingivitis by adding antimicrobial agents, such as fluorides, chlorhexidine, and stannous chloride, and achieved considerable success.^[28]^ However, biofilm bacteria usually exhibit up to 1000-fold greater resistance to antimicrobial agents compared to planktonic cells.^[29]^ Recent knowledge has highlighted the role of oral microbiota in the development of periodontal disease. Maintaining a balanced plaque microbiota is identified as a key strategy for improving periodontal disease.^[30]^ During the transition from health to periodontal disease, the subgingival microbial community shifts towards a pathogenic state through changes in species composition and abundance.^[30]^ In this study, observed species index and Chao index were reduced significantly after using the OPTIMEALTH® OR toothpaste, while the changes in the Shannon index and beta diversity were not significant, indicating that the effects of OPTIMEALTH® OR toothpaste mainly involved reducing the richness of microbial populations rather than altering their composition. Nonetheless, we found that the relative abundance of *Veillonella parvula* and *Prevotella denticola* was reduced after using OPTIMEALTH® OR toothpaste for 4 weeks. Several dominant species contribute to the periodontal disease. *V parvula* is one of the early colonizers of biofilm formation, contributing to the progression of aggressive periodontitis and root caries.^[31–34]^ *P denticola* is commonly observed in patients suffering from aggressive periodontitis.^[35]^ It has been reported that the symbiotic relationship between *P. denticola* and *Streptococcus mutans* contributes to the enhanced virulence of plaque biofilms.^[36]^ The relatively reduction of these 2 periodontal disease-related species, combined with the reduction in alpha diversity, implicated the improvement of microbial community ecology using OPTIMEALTH® OR toothpaste. *Porphyromonas gingivalis* is a pivotal pathogen in the development of periodontitis.^[37]^ *P gingivalis* exerts a microbial-driven role in the transition from periodontal health to disease by upregulating virulence genes.^[30]^ Paradoxically, the sequencing results indicate that *P. gingivalis* was significantly upregulated after the application of any toothpaste, which may be attributed to some adverse effects of the blank matrix toothpaste. However, notably, a more significant upregulation was observed with toothpaste not supplemented with OPTIMEALTH® OR. In short, this study found that OPTIMEALTH® OR toothpaste improved clinical GI markers (particularly PI and BOP%), possibly due to the regulation of the oral microbiota, especially when considering that several species closely associated with periodontal disease (*V parvula* and *P denticola*) were reduced by using OPTIMEALTH® OR toothpaste.

Our results support the beneficial effects of postbiotics on human microflora and health. Postbiotics are defined as the inanimate microorganisms and/or their components that confer a health benefit on the host.^[30]^ As functional fermentation compounds, besides offering health benefits similar to probiotics and prebiotics, postbiotics can also overcome certain technical challenges, such as eliminating the need for microbial colonization, extending shelf life, and facilitating packaging and transportation.^[38]^ Studies have shown that probiotics can help prevent periodontal disease by reducing the quantity of periodontal pathogens and preventing the formation of dental plaque by lowering saliva pH and generating antioxidants.^[39]^ Lin and his colleague demonstrated that using a food additive in the form of an oral postbiotic or heat-killed probiotic lozenge could significantly improve oral immunity, suppress the proliferation of oral pathogens, and promote an increase in beneficial oral microbiota.^[40]^ Probiotics exert their functional mechanism through the production of compounds such as bacteriocins, organic acids, fatty acids, and hydrogen peroxide.^[39]^ These microbial metabolic substances, or postbiotics, may have better and more efficient effects than probiotics and prebiotics in application.^[41]^ The test sample of this study, OPTIMEALTH® OR, is a microbial fermentation product with over 500 compounds, of which more than 300 have been identified (data not displayed). However, how these active compounds specifically function on oral health remains to be further explored. Cosmetics and personal care products are mostly directly applied to the human epidermis or mucosal tissue, where postbiotics exert their microbial regulatory effects. Therefore, our positive results may support incorporating prebiotics into cosmetics and personal care products.

Here are the limits of the study. This study focuses on the changes in dental plaque and aims to characterize the alterations in oral microbiota before and after using OPTIMEALTH® OR toothpaste. Initially, participants were recruited based on the PI (see materials and methods), which may have resulted in the outcomes not accurately reflecting the improvement of gingivitis. Furthermore, the short duration of the study, lasting only 4 weeks, may have limited the comprehensive reflection of certain indices, such as the GI and BOP%. Additionally, the limited number of participants, wide age range, and imbalance in gender ratio could also restrict the conclusions drawn from this study. In order to thoroughly evaluate the effect of OPTIMEALTH® OR toothpaste on gingivitis, future research should consider a more comprehensive trial design, including baseline GI and/or BOP levels as inclusion criteria, longer duration of participant follow-up, incorporation of negative and positive controls, concentration gradient testing, post-treatment follow-ups, and so on.

## 5. Conclusions

In summary, using toothpaste with 2% OPTIMEALTH® OR could effectively decrease plaque accumulation and modulate plaque bacteriota. It had the potential to inhibit gingivitis, although further research is still needed with more subjects.

## Author contributions

**Conceptualization:** Yimin Fu, Hui Chai.

**Data curation:** Yimin Fu, Yong Yang.

**Formal analysis:** Yimin Fu, Yong Yang, Keyun Mu, Yuye Zhou.

**Writing – original draft:** Yimin Fu, Hui Chai.

**Writing – review & editing:** Yimin Fu, Hui Chai.

**Investigation:** Keyun Mu, Hui Chai.

**Visualization:** Yuye Zhou.
